# Predictors of cervical cancer screening among Kenyan women: results of a nested case-control study in a nationally representative survey

**DOI:** 10.1186/s12889-018-6054-9

**Published:** 2018-11-07

**Authors:** Anne Ng’ang’a, Mary Nyangasi, Nancy G Nkonge, Eunice Gathitu, Joseph Kibachio, Peter Gichangi, Richard G Wamai, Catherine Kyobutungi

**Affiliations:** 1 NCD Division National Cancer Control Program, Ministry of Health, Nairobi, Kenya; 2 Division of Pharmacy, Kenyatta National Hospital, Nairobi, Kenya; 3 Division of Non Communicable Diseases, Ministry of Health, Nairobi, Kenya; 4 The Institute of Global Health, Faculty of Medicine, University of Geneva (UNIGE), Geneva, Switzerland; 5 Department of Human Anatomy, University of Nairobi, Nairobi, Kenya; 6 Department of Cultures, Societies and Global Studies, North Eastern University, Massachusetts, USA; 7 African Population and Health Research Centre, Nairobi, Kenya

**Keywords:** Cervical cancer, Screening, Health behaviors, Kenya

## Abstract

**Background:**

Cervical cancer is a major public health concern in Kenya. It is the leading cause of cancer morbidity and mortality among women. Although screening is an effective prevention method, uptake is low among eligible women. Little is known about predictors of cervical cancer screening uptake. This study explored relationship between uptake of cervical cancer screening, socio-demographic, behavioral and biological risk factors.

**Methods:**

Nested case-control study within STEPS survey, a population-based cross-sectional household survey conducted between April and June 2015.Cases were women who had undergone cervical cancer screening and controls were unscreened women. Study participants were women eligible for cervical cancer screening (30–49 years). Variables included socio-demographic; behavioral risk factors such as physical activity, tobacco and alcohol use diet and biological factors like diabetes and hypertension. Outcome of interest was cervical cancer screening. Data analysis was done using STATA version 14. Logistic regression model was used to assess relationship between cervical cancer screening and socio-demographic, behavioral and biological risk factors.

**Results:**

Of 1180 women interviewed, 16.4% (*n* = 194) had been screened for cervical cancer. Of unscreened women (*n* = 986), 67.9% were aware of cervical cancer screening. Higher screening rates were observed in more educated women (25.2%), highest income quintile (29.6%) and living in urban areas (23%) than in women with no formal education (3.2%), poorest (3.6%) and living in rural areas (13.8%). Younger women (35–39) and those with low High-density lipoprotein (HDL) were less likely to be screened [OR = 0.56; 95% CI = (0.34, 0.93); *p*-value = 0.025] and [OR = 0.51; 95% CI = (0.29, 0.91); p = value 0.023] respectively. Self-employed women, those in the fourth wealth quintile, binge drinkers, high sugar consumption and insufficient physical activity were more likely to be screened [OR 2.55 (1.12, 5.81) *p* value 0.026], [OR 3.56 (1.37, 9.28) p value 0.009], [OR 5.94 (1.52, 23.15) p value 0.010], [OR 2.99 (1.51, 5.89) p value 0.002] and [OR 2.79 (1.37, 5.68) p value 0.005] respectively.

**Conclusion:**

Uptake of cervical cancer screening is low despite high awareness. Strategies to improve cervical cancer screening in Kenya should be implemented with messages targeting persons with both risky and non-risky lifestyles especially younger women with no formal education living in rural areas.

## Background

Cancer is the second leading cause of death worldwide accounting for 8.7 million deaths globally in 2015 and 17.5 million new diagnoses with a 33% increase in cases between 2005 and 2015 [1]. Among this group of diseases, cervical cancer has in recent years been the leading cause of cancer deaths worldwide [2–4] with 239,000 deaths and 526,000 diagnoses in 2015 and most deaths being in sub-Saharan Africa (SSA) [1]. The global distribution and rising prevalence of cancer shows a worrisome ‘cancer divide’ where survival rates are low and outcomes poor among socioeconomically disadvantaged populations due to weak health systems, poor levels of education, and low awareness and screening coverages [5–10]. While cervical cancer is primarily caused by the sexually transmitted human papillomavirus (HPV) [11, 12], further variation is caused by differential prevalence levels of the infectious agent with SSA carrying the greater overall burden [13–16].

With close to 99,000 new cases and 57,400 deaths in 2012 alone [3], cervical cancer is emerging as a major public health problem in SSA [17, 18] where close to 90% of 443,000 projected annual global cervical cancer deaths by 2030 will occur [18]. This is due to population aging and growth, as well as increased prevalence of risk factors including those associated with social and economic transition [19–23]. Furthermore, as a sexually transmitted infection, behavioral factors are critical in the increased risk for HPV in high HIV prevalence setting in SSA where these infections are likely synergistic [24–29].

Although these are worrisome trends, it is noteworthy that attention to cervical cancer has been increasing in the past decade with major milestones and global commitments. These include: the development of three safe and effective HPV vaccines since 2006 [30–32], the 2009 World Health Organization’s (WHO) position paper on HPV vaccines [33], 2011 Political Declaration on non-communicable diseases (NCDs) at the United Nations High-Level Meeting [34], the 2013 WHO *Global action plan for the prevention and control of NCDs 2013–2020* [35]*,* the Global Task Force on Expanded Access to Cancer Care and Control in Developing Countries [36], the commitment by GAVI Alliance in 2013 to support cervical cancer immunization at drastically reduced prices of $4.50 per dose for Gardasil vaccine and $4.60 per dose for Cevarix vaccine to qualifying countries [37], the 2014 WHO guidelines on cervical cancer screening [38] and in 2015 as reflected in the third Sustainable Development Goal (SDG) to reduce premature mortality due to NCDs by one-third by 2030 [39].

Despite these milestones and the high global burden of cervical cancer on SSA, screening and vaccine coverage remain overall very low [40–42] except for a few countries like Rwanda which reached 93% of adolescent girls in grade six in 2011 [43]. Cervical cancer is preventable and curable in early stages through primary, secondary and tertiary interventions involving vaccination, early diagnosis and treatment [38]. In developed countries, screening and immunization has led to a marked reduction in incidence and mortality from cervical cancer [44, 45]. Drastically increasing screening availability will be a first crucial step to beginning to tackle cervical cancer in SSA. Although countries have started exploring strategies to address awareness, prevention, screening and immunization [41, 46–48] and research in these areas is increasing [49, 50], much remains to be done.

In Kenya, cervical cancer is the second most common cancer in women but the most common cause of cancer deaths [2, 51]. While accuracy of numbers are uncertain due to lack of a national cancer registry, of 2,354 women diagnosed in 2006 in the Nairobi Cancer Registry 65% died [52]. The latest (2014) Kenya Demographic Health Survey (KDHS) shows a screening uptake of only 14% among women 30–49 years of age [53]. Although the Kenya government has developed a national cervical cancer prevention strategic plan [51] as well as conducted the vaccination pilot, rollout of the program has not yet commenced. Several studies on awareness and vaccine acceptability have been conducted in the country but only in some localized parts of the country such as Eldoret in the west [54–57], Nyanza [58, 59] and in Nairobi [60, 61]. A study from the Kitui pilot assessed primary school teachers’ knowledge and acceptability of HPV vaccine [62]. These studies reveal high awareness and vaccine acceptability but also several factors contributing to the low screening uptake including lack of access to health facilities, cost, shortage of necessary supplies, inadequate and untrained staff, fear and perceived discomfort, long waiting time and lack of sufficient knowledge on the disease process. Many of these factors are also demonstrated in other low-and mid-income countries [10, 42, 63–66].

While some of the studies in Kenya assess behavioral risk factors and cervical cancer screening, it is not clear whether and which health behaviors can predict uptake of cervical cancer screening and no national-level data existed prior to the current study. The objective of this study was to determine the level of awareness of cervical cancer screening in women, examine the socio-demographic factors affecting cervical cancer screening, describe health behaviors in screened women and unscreened women and investigate whether biological risk factors predict uptake of cervical cancer screening using the first nationally representative survey on NCDs.

## Methods

The nested unmatched case-control study design included all women between 30 and 49 years of age who had been recruited during the STEPS survey [67], a nationally representative survey conducted between May and June 2015. Written informed consent was sought from the selected individuals and confidentiality was maintained by all personal identifiers delinked by coding. The study protocol was approved by Kenya Medical Research Institute’s Ethics Review Committee (SSC No. 2607).

Variables analyzed were behavioral risk factors as defined by the WHO STEPS instrument: current smoking habits, current alcohol consumption habits, diet and physical activity; socio-demographic factors such as age, residence, marital status, education level, wealth quintile and employment status and biological risk factors such as diabetes and, hypertension. The dependent variable was the screening status.

In the STEPS report, hypertension is defined by WHO as a systolic blood pressure ≥ 140 mmHg and/or diastolic blood pressure ≥ 90 mmHg and a fasting blood glucose of > 7 mmol/l is diagnostic of diabetes mellitus while a level of 6.1–7 mmol/l is known as impaired fasting glycaemia (pre-diabetic state); Descriptive analysis included frequencies and percentages of the sociodemographic variables. Prevalence of current smoking habits, current alcohol consumption habits, unhealthy diet and physical inactivity in the screened and unscreened women were also calculated.

We considered all the known cervical cancer risk factors in our data for analysis. Bivariate logistic regression models were run to assess the bivariate relationship between the different risk factors and the outcome of interest, cervical cancer screening. We then included all the risk factors of interest and those which had a *p*-value of less than 0.25 in the regression model. For the bivariate analyses comparing the study variables between screened and unscreened women, unpaired t-test and Chi squared test were used for continuous variables and categorical variables, respectively. Odds ratio and its 95% confidence interval were estimated and computed for each significant categorical factor using binary logistic regression. Conditional logistic regression analysis was performed to analyze the association between all categorical factors and screening status. All analyses were performed with STATA version 14.

## Results

### Sociodemographic characteristics, screening awareness and behaviors

Descriptive data are presented in Table 1. More than half (64.0%) of the women interviewed were between 30 and 39 years. Almost all (92.9%) of them were either currently married or formerly married. Most (83.8%) of them had received formal education in primary, secondary or tertiary levels of education. Homemakers and self-employed women constituted more than three quarters (79.6%) of the participants. Participants were evenly distributed in the wealth quintile bands despite majority (71.6%) of them living in the rural areas.

**Table 1:** Sociodemographic characteristics, screening awareness and behaviors by unscreened and screened

Characteristic	Cervical Cancer Screened			
	Unscreened (%)	Screened (%)	Total (%)	Uncorrected Pearson Chi2
Age groups				
30–34	334 (82.8)	69 (17.2)	403 (34.2)	chi2(3) = 15.26, p-value = 0.025
35–39	312 (88.6)	40 (11.4)	352 (29.8)	
40–44	195 (76.9)	59 (23.1)	253 (21.4)	
45–49	146 (85.0)	26 (15.0)	171 (14.5)	
*Total*	*986 (83.6)*	*194 (16.4)*	*1180 (100)*	
Marital status				
Not married	68 (82.1)	15 (17.9)	83 (7.0)	chi2(2) = 0.96, *p* = 0.753
Married	761 (84.2)	143 (15.8)	903 (76.5)	
Formerly married	157 (81.5)	36 (18.5)	193 (16.4)	
*Total*	*986 (83.6)*	*194 (16.4)*	*1180 (100)*	
Education level				
No formal education	185 (96.8)	6 (3.2)	192 (16.3)	chi2(2) = 44.11, *p* < .000
Primary incomplete	273 (86.1)	44 (13.9)	317 (26.9)	
Primary complete	267 (82.8)	56 (17.2)	323 (27.4)	
Secondary and above	261 (74.8)	88 (25.2)	349 (29.6)	
*Total*	*986 (83.6)*	*194 (16.4)*	*1180 (100)*	
Occupation				
Unemployed	76 (87.4)	11 (12.6)	87 (7.4)	chi2(5) = 24.78, *p* = 0.006
Employed	111 (72.9)	41 (27.1)	152 (12.9)	
Homemaker	420 (88.5)	55 (11.5)	475 (40.3)	
Non-paid/volunteer	1 (100.0)	0 (0.0)	1 (0.1)	
Self-employed	378 (81.4)	86 (18.6)	464 (39.3)	
Student	0 (25.0)	0 (75.0)	0 (0.0)	
*Total*	*986 (83.6)*	*194 (16.4)*	*1180 (100)*	
Wealth band				
Poorest	257 (96.4)	9 (3.6)	266 (22.5)	chi2(4) = 75.3041, *p* < 0.000
Second	260 (89)	32 (11)	292 (24.7)	
Middle	198 (80.3)	49 (19.7)	247 (20.9)	
Fourth	137 (74.7)	46 (25.4)	183 (15.5)	
Richest	135 (70.4)	57 (29.6)	192 (16.3)	
*Total*	*986 (83.6)*	*194 (16.4)*	*1180 (100)*	
Residence				
Rural	729 (86.2)	117 (13.8)	845 (71.6)	chi2(1) = 14.78, p = 0.006
Urban	258 (77.0)	77 (23.0)	335 (28.4)	
*Total*	*986 (83.6)*	*194 (16.4)*	*1180 (100)*	
Awareness of cervical cancer screening				
No	573 (99.2)	5 (0.8)	578 (49.6)	chi2(1) = 206.71, p < 0.000
Yes	399 (67.9)	189 (32.1)	588 (50.4)	
*Total*	*972 (83.4)*	*194 (16.6)*	*1166 (100)*	
Current tobacco use				
No	948 (83.3)	190 (16.7)	1138 (96.4)	chi2(1) = 2.60, *p* = 0.103
Yes	39 (92.6)	3 (7.4)	42 (3.6)	
*Total*	*986 (83.6)*	*194 (16.4)*	*1180 (100)*	
Episodic alcohol drinking				
No alcohol	952 (84.4)	176 (15.6)	*1128 (95.8)*	chi2(2) = 12.16, *p* = 0.134
Binge drinking	18 (66.1)	9 (33.9)	28 (2.4)	
Non-heavy drinking	16 (66.0)	8 (34.0)	24 (2)	
*Total*	*984 (83.6)*	*194 (16.4)*	*1178 (100)*	
Inadequate fruits and vegetables				
No	691 (80.8)	164 (19.2)	855 (72.5)	chi2(1) = 17.28, *p* = 0.001
Yes	296 (90.9)	30 (9.1)	325 (27.5)	
*Total*	*986 (83.6)*	*194 (16.4)*	*1180 (100)*	
Excess sugar				
No	168 (90.7)	17 (9.3)	186 (15.8)	chi2(1) = 8.10, *p* = 0.024
Yes	818 (82.3)	176 (17.7)	994 (84.2)	
*Total*	*986 (83.6)*	*194 (16.4)*	*1180 (100)*	
Actual intake of salt				
Low salt (7 and below	823 (83.2)	167 (16.9)	990 (83.9)	chi2(1) = 0.08, *p* = 0.840
High (above 7)	163 (85.9)	27 (14.1)	190 (16.1)	
*Total*	*986 (83.6)*	*194 (16.4)*	*1180 (100)*	
Physical activity				
Sufficient	932 (84.4)	173 (15.7)	1105 (93.6)	chi2(1) = 7.15, *p* = 0.020
Insufficient	55 (72.6)	21 (27.5)	75 (6.4)	
*Total*	*986 (83.6)*	*194 (16.4)*	*1180 (100)*	
Diabetic				
No	900 (83.7)	175 (16.3)	1075 (97.1)	chi2(1) = 1.60, *p* = 0.332
Yes	24 (75.3)	8 (24.7)	32 (2.9)	
*Total*	*924 (83.5)*	*183 (16.5)*	*1107 (100)*	
Hypertensive				
No	296 (86.3)	47 (13.7)	342 (29.4)	chi2(1) = 2.24, *p* = 0.233
Yes	680 (82.8)	141 (17.2)	822 (70.6)	
*Total*	*976 (83.8)*	*188 (16.2)*	*1164 (100)*	

Half of the women (50.4%) were aware of cervical cancer screening but only 16.4% had ever been screened. Of the unscreened women, 67.9% were aware of cervical cancer screening. Higher screening rates were observed in more educated women (25.2%), women in the highest income quintile (29.6%) and women living in urban areas (23%) than in women with no formal education (3.2%), women in the lowest income quintile (3.6%) and in women living in rural areas (13.8%).

Current tobacco users and alcohol drinkers were a small proportion of the women interviewed. A higher proportion (84.2%) of participants consumed excess sugar compared to those who had inadequate fruit and vegetables intake (27.5%), high salt intake (16.1%) and insufficient physical activity. (6.4%). Higher screening rates were found in tobacco non-users (16.7%), binge drinkers (33.9%), women with adequate fruits and vegetables intake (19.2%), excess sugar intake (17.7%), low salt intake (16.9%) and insufficient physical activity (27.5%) than in current tobacco users (7.4%), alcohol non-drinkers (15.6%), women with inadequate fruits and vegetables intake (9.1%), less sugar intake (9.3%), high salt intake (14.1%) and sufficient physical activity (15.7%).Higher screening rates were noticed in women with diabetes (24.7%) and hypertension (17.2%) than in women without diabetes (16.3%) and without hypertension (13.7%).

### Determinants of uptake of cervical cancer screening

According to Table 2, younger women (35–39) and those with low HDL were less likely to be screened [OR 0.56 (0.34, 0.93) *p* value 0.025] and [OR 0.51 (0.29, 0.91) *p* value 0.023] respectively. Self-employed women, those in the fourth wealth quintile, binge drinkers, women with high sugar consumption and insufficient physical activity were more likely to be screened [OR 2.55 (1.12, 5.81) *p* value 0.026], [OR 3.56 (1.37, 9.28) *p* value 0.009], [OR 5.94 (1.52, 23.15) *p* value 0.010], [OR 2.99 (1.51, 5.89) *p* value 0.002] and [OR 2.79 (1.37, 5.68) *p* value 0.005] respectively.

**Table 2:** Determinants of uptake of cervical cancer screening

Cancer screen	Crude Odds Ratio		Adjusted Odds Ratio	
	OR (95% CI)	p-value	OR (95% CI)	p-value
Age group				
30–34	1.00		1.00	
35–39	0.62 (0.41, 0.94)	0.024	0.54 (0.32, 0.90)	0.018
40–44	1.45 (0.98, 2.14)	0.063	1.50 (0.90, 2.52)	0.121
45–49	0.85 (0.52, 1.39)	0.517	1.00 (0.53, 1.88)	0.988
Marital status				
Not married	1.00			
Married	0.86 (0.48, 1.55)	0.618	1.48 (0.73, 3.02)	0.275
Formerly married	1.04 (0.53, 2.02)	0.912	1.58 (0.70, 3.59)	0.270
Education level				
No formal education	1.00		1.00	
Primary incomplete	4.80 (2.03, 11.34)	0.000	0.83 (0.28, 2.53)	0.749
Primary complete	6.21 (2.65, 14.51)	0.000	0.63 (0.20, 1.99)	0.426
Secondary and above	10.03 (4.35, 23.12)	0.000	0.76 (0.24, 2.46)	0.653
Occupation				
Unemployed	1.00		1.00	
Employed	2.59 (1.25, 5.36)	0.010	1.76 (0.69, 4.52)	0.236
Homemaker	0.91 (0.45, 1.81)	0.783	2.24 (0.99, 5.10)	0.054
Non-paid/volunteer	1 (empty)		1 (empty)	
Self-employed	1.59 (0.81, 3.12)	0.180	2.55 (1.12, 5.81)	0.026
Student	20.92 (0.02, 22921.15)	0.395	545.08 (0.34, 873199.6)	0.094
Wealth band				
Poorest	1.00		1.00	
Second	3.35 (1.59, 7.04)	0.001	1.48 (0.59, 3.74)	0.403
Middle	6.63 (3.23, 13.62)	0.000	2.26 (0.92, 5.59)	0.076
Fourth	9.18 (4.43, 19.02)	0.000	3.12 (1.19, 8.21)	0.021
Richest	11.39 (5.55, 23.36)	0.000	2.19 (0.76, 6.31)	0.148
Residence				
Rural	1.00		1.00	
Urban	1.87 (1.35, 2.57)	0.000	0.82 (0.51, 1.33)	0.421
Heard of cervical cancer				
No	1.00		1.00	
Yes	59.47 (23.29, 151.87)	0.000	66.75 (23.77, 187.43)	0.000
Tobacco use				
No	1.00		1.00	
Yes	0.40 (0.12, 1.27)	0.119	0.71 (0.12, 4.42)	0.717
Alcohol consumption				
No alcohol	1.00		1.00	
Binge drinking	2.78 (1.25, 6.19)	0.012	5.94 (1.52, 23.15)	0.010
Non-heavy drinking	2.78 (1.18, 6.60)	0.020	3.78 (0.96, 14.84)	0.057
Inadequate fruits and vegetables				
No	1.00		1.00	
Yes	0.42 (0.28, 0.64)	0.000	0.79 (0.46, 1.37)	0.404
Excess sugar				
No	1.00		1.00	
Yes	2.10 (1.25, 3.54)	0.005	3.17 (1.61, 6.21)	0.001
Salt intake				
Low	1.00		1.00	
High (above 7)	0.81 (0.52, 1.26)	0.344	1.17 (0.65, 2.12)	0.601
Physical activity				
Sufficient	1.00		1.00	
Insufficient	2.04 (1.20, 3.47)	0.009	2.76 (1.34, 5.67)	0.006
Diabetes				
No	1.00		1.00	
Yes	1.68 (0.74, 3.81)	0.211	1.37 (0.46, 4.13)	0.574
Hypertension				
No	1.00		1.00	
Yes	1.31 (0.92, 1.88)	0.135	0.99 (0.64, 1.55)	0.974
HDL				
Normal	1.00		1.00	
Low	0.67 (0.41, 1.09)	0.104	0.50 (0.28, 0.88)	0.016

## Discussion

This study results from the first nationally representative study of NCDs in Kenya. The study sought to assess the level of awareness of cervical cancer screening among women, describe health behaviors in screened women and unscreened women and determine predictors of cervical cancer screening. In 2015, 16.4% of eligible Kenyan women had been screened for cervical cancer, a marginally higher figure than the 14% shown in the 2014 KDHS [53]. Awareness of cervical cancer screening was high with 67.9% of unscreened women being aware, a lower level than in the KDHS where it was 76%. The gap between awareness and uptake of cervical cancer screening is consistent with other studies. In a study done in Uganda, only 4.8% of women had undergone screening for cervical cancer despite high levels of knowledge about cervical cancer and its risk factors [68].

Higher screening rates were seen in older, more educated, richer women and those living in urban areas. This is similar to a study done in Tanzania [69]. Older women are more likely to have interacted with the health system longer and therefore more likely to have undergone cervical cancer screening. A study in France found high screening rates among younger women aged 25–35 year [70]. The explanation for this was the screening services provided during antenatal visits. This calls for integration of cervical cancer services within the Kenya health system to avoid missed opportunities. While this is noted in various national health documents notably the National Cervical Cancer Prevention Program [52], the current practice shows a lack of cervical cancer services across the public health system [53]. Access to health services in rural areas has been cited as a barrier in other African setting [69] and could explain the higher screening rates among urban women. Even though cervical cancer screening is free in the public health sector in Kenya, additional costs such as transport may explain low screening rates among the women in lower wealth quintiles. Programs to increase cervical cancer screening should factor in hidden costs such as transport or lost earnings as women seek screening services especially in asymptomatic phase.

Our study had interesting findings regarding health behaviors. There were higher rates of screening among tobacco non-users, those with adequate fruit and vegetable intake and low salt intake. We also found high rates of screening among binge drinkers, those with excess sugar intake and those with insufficient physical activity. These results are consistent with another study [70] that showed that screening uptake is not predictable based on health behaviors. This suggests that primary prevention programs should target all populations including those with healthy lifestyles and not just those with risky lifestyles.

Our paper also looked at screening uptake among women with diabetes and hypertension. Women with diabetes and hypertension had higher screening rates than those without. These findings are in contrast to existing literature except for hypertension. A study in the US showed that women with hypertension had increased odds of screening for breast cancer than non-hypertensives but no difference for cervical cancer screening [71]. The explanation for higher screening rate in the Kenyan women could be due to frequent contact with health care though other literature suggests quality of chronic disease care explains uptake of cancer screening and not necessarily frequent visits to health facilities [72].

This study had limitations. The uptake of cervical cancer screening was based on self-reports, with possible social desirability bias or recall bias. There was no data collected on frequency of screening, method used or where the screening was conducted. We are therefore not able to examine the most frequently used method or venue of screening. We cannot explore if the cervical cancer screening introduced in public health facilities is functional or not. Further studies are required to determine where women receive their cervical cancer screening and methods used for screening to better address the barriers to access or uptake of cervical cancer screening.

## Conclusion

In conclusion, the study showed that among women aged 30–49 years, in terms of socio-demographic characteristics, those that were most likely to be screened were those in the age group 40–44 years, formerly married, the more educated women, and self-employed, richest women and living in urban areas.

For the behavioral risk factors, higher screening rates were found in tobacco non-users, alcohol binge drinkers, women with adequate fruits and vegetables intake, excess sugar intake, low salt intake and insufficient physical activity.

For biological risk factors, higher screening rates were observed in diabetics, hypertensives and in women with normal HDL.

As Kenya prepares to roll out the national HPV vaccination program in 2019, there is need to increase public awareness on the need for cervical cancer screening and develop strategies on the same to ensure increased screening uptake, early detection and better treatment outcomes. Advocacy initiatives should focus on younger women aged 35-39 years, and persons with risky as well as non-risky lifestyles.

## Abbreviations

HDL: High density lipoprotein
HPV: Human Papilloma virus
LDL: Low density lipoprotein
MOH: Ministry of health
NCD: Non-communicable diseases
